# Transthyretin amyloid cardiomyopathy in women: frequency, characteristics, and diagnostic challenges

**DOI:** 10.1007/s10741-020-10010-8

**Published:** 2020-08-14

**Authors:** Marianna Bruno, Adam Castaño, Arianna Burton, Justin L. Grodin

**Affiliations:** 1 Medical Affairs, Pfizer Inc., New York, NY USA; 2 Department of Internal Medicine, University of Texas Southwestern Medical Center, Dallas, TX USA; 3 Clinical Heart and Vascular Center, University of Texas Southwestern Medical Center, 5323 Harry Hines Blvd, Suite E5.310F, Dallas, TX 75390-8830 USA

**Keywords:** Transthyretin amyloid cardiomyopathy, Heart failure, Epidemiology, Diagnosis, Sex

## Abstract

Transthyretin amyloid cardiomyopathy (ATTR-CM) is a progressive, life-threatening disease characterized by deposition of insoluble amyloid fibrils in the myocardium, resulting in cardiac structural and functional abnormalities and ultimately heart failure. Disease frequency is reportedly lower in women than men, but sex-related differences have not been well established. We conducted a systematic literature review (SLR), based on PRISMA-P guidelines and registered with PROSPERO, to assess whether the epidemiology and clinical presentation of ATTR-CM differ between women and men. MEDLINE, Embase, and Cochrane databases and selected conference proceedings were searched (August 16, 2019) to identify observational and clinical studies reporting sex-specific data for patients with wild-type or hereditary ATTR-CM. Of 193 publications satisfying final eligibility criteria, 69 studies were included in our pooled analysis. Among the 4669 patients with ATTR-CM analyzed, 791 (17%) were women, including 174 (9%), 366 (29%), and 251 (18%) in studies of wild-type, hereditary, and undefined ATTR-CM, respectively. Data available on disease characteristics were limited and very heterogeneous, but trends suggested some cardiac structural/functional differences, i.e., lower interventricular septal and posterior wall thickness and left ventricular (LV) end diastolic diameter, and higher LV ejection fractions, in women versus men across ATTR-CM subtypes. Because LV wall thickness > 12 mm is generally the suggested threshold for ATTR-CM diagnosis in both sexes, smaller cardiac anatomy in women with the disease may lead to underdiagnosis. Additional research and studies are needed to elucidate potential disparities between sexes in ATTR-CM frequency, clinical characteristics, and underlying biological mechanisms. This study was registered within the International Prospective Register of Systematic Reviews (PROSPERO) database of the University of York (CRD42019146995).

**Electronic supplementary material:**

The online version of this article (10.1007/s10741-020-10010-8) contains supplementary material, which is available to authorized users.

## Introduction

Transthyretin amyloid cardiomyopathy (ATTR-CM) is a progressive, often underdiagnosed and misdiagnosed disease caused by the extracellular deposition of misfolded transthyretin protein (TTR) that forms insoluble amyloid fibrils in organs and tissues [1]. Infiltration of the myocardium by amyloid fibrils ultimately results in life-threatening cardiac structural and functional abnormalities and heart failure [1–3]. The condition may be inherited as an autosomal dominant trait associated with mutations of the transthyretin gene *TTR* (hereditary ATTR-CM) or caused by age-dependent, non-mutational mechanisms (wild-type ATTR-CM). The prevalence of hereditary ATTR-CM varies by region due to fluctuating geographic distribution of *TTR* mutations; the Val122lle mutation, the most common in the USA, has been reported in approximately 3–4% of African Americans [4]. Among hospitalized patients with heart failure and preserved ejection fraction, older than 60 years of age, with left ventricular wall thickness of 12 mm or greater on scintigraphy, the prevalence of wild-type ATTR-CM was 13% [5]. The frequency of ATTR-CM, particularly the wild-type form, is reportedly lower in women than men [6–11], and the female sex has been proposed as a protective factor [1]. However, the higher proportions (over 50%) of women with wild-type ATTR observed in some study populations [5, 12] provide theoretical support that ATTR-CM may be further underdiagnosed and/or misdiagnosed in women because of sex-related differences in clinical presentation or disease characteristics [7, 13].

Given the availability of definitive treatments for ATTR-CM, early diagnosis in women, as in all patients, is critical to slowing disease progression and improving outcomes before overt multi-organ dysfunction ensues [13–17]. Despite the multiple diagnostic imaging options currently available, including advanced techniques such as nuclear scintigraphy that allow non-invasive assessment in select populations, and expert consensus recommendations for their use in ATTR-CM [18, 19], diagnosis of the disease remains a challenge. Clinicians’ awareness of cardiac and non-cardiac clinical signs and symptoms, such as unexplained increases in left ventricular (LV) wall thickness on echocardiography, atrial fibrillation, bilateral carpal tunnel syndrome, and lumbar spinal stenosis, should raise suspicion of ATTR-CM and help facilitate prompt diagnosis [7]. However, the presumption of male predominance of wild-type and hereditary ATTR-CM may lower clinical suspicion of the disease when such “red flags” are observed in women.

We conducted a systematic literature review (SLR) to examine the epidemiology of ATTR-CM in women, to evaluate whether the clinical presentation and characteristics of the disease differ between women and men, and to explore potential reasons for these differences.

## Methods

The SLR was designed to identify studies that reported sex-specific findings for patients with ATTR-CM, including wild-type and hereditary subtypes. The SLR was conceived and conducted according to the Preferred Reporting Items for Systematic Reviews and Meta-Analyses Protocol (PRISMA-P) guidelines [20] and was registered within the International Prospective Register of Systematic Reviews (PROSPERO) database of the University of York (CRD42019146995) [21].

### Search strategy and data sources

A comprehensive search strategy was developed to find studies reporting data on women with ATTR-CM in the published literature. Multiple search terms for transthyretin amyloidosis and variations of the wild-type and hereditary subtypes were used in combination with terms for cardiomyopathy and women using the Boolean operator “AND” (Supplementary Table 1). Searches were conducted for studies published in English through August 16, 2019, based on electronic searches of the following databases: MEDLINE^®^, Embase^®^, Cochrane Central Register of Controlled Trials, and Cochrane Database of Systematic Reviews. A manual cross-referencing search was also conducted of the PubMed^®^ database. Reference lists of the published literature identified in the database searches, as well as relevant review articles, were manually searched to identify publications that may not have appeared in the original electronic searches. In addition, to capture the most recent and relevant studies on this topic that are not yet published, manual searches were conducted of proceedings from the following conferences: the European Society of Cardiology, Heart Failure Society of America, and International Society of Amyloidosis.

### Study screening, selection, and data extraction

**Fig. 1:**
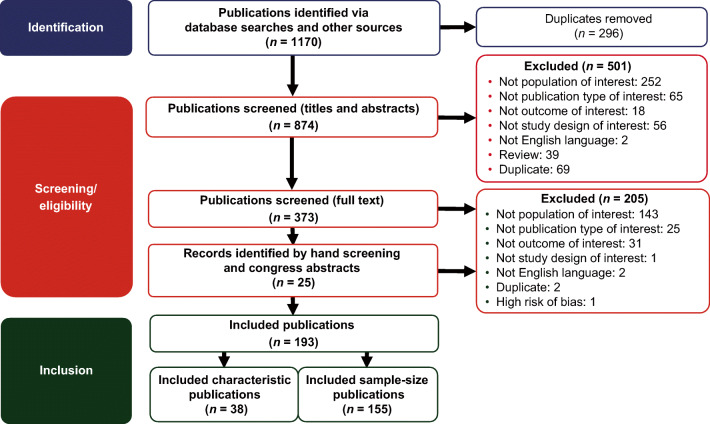
Flow of publications/studies through the search and screening process

The flow of publications/studies through the search and screening process is summarized in Fig. 1. After removal of duplicate citations in the initial searches, publication titles and abstracts were assessed against inclusion/exclusion criteria in the first of 2 rounds of screening by 2 independent reviewers. In the second screening round, the reviewers examined complete texts of the publications for eligibility. If agreement on discrepancies between the 2 reviewers could not be reached, a third senior investigator reached a final decision. Data from the included publications were extracted by one reviewer and validated by the second reviewer for quality assurance, then reported in pre-specified data-extraction tables. Excluded studies were documented along with the reason for their exclusion.

Based on the PICOS (Population, Intervention, Comparison, Outcome, Study design) framework [22], study inclusion criteria encompassed the following: (i) original studies of adult women with ATTR-CM, including subpopulations of women with wild-type and hereditary disease subtypes, (ii) interventional or non-interventional studies with or without comparator, (iii) studies reporting sex-specific data (e.g., demographic characteristics, ATTR genotype, clinical measures, biomarkers, imaging, incidence, frequency, mortality, comorbidities, and hormonal status), and (iv) controlled clinical trials (randomized and non-randomized) and observational studies (prospective and retrospective). Diagnostic criteria used to identify patients with ATTR-CM were based primarily on recommendations of the 2019 Multi-societal Expert Consensus Committee (Supplementary Table 2) [23]. The hereditary subtype of ATTR-CM was confirmed via *TTR* genopositivity established by DNA analysis or mass spectrometry.

### Pooled analysis

Findings from selected included studies were pooled to analyze the proportions of women and men who had wild-type ATTR-CM, hereditary ATTR-CM, and undefined ATTR-CM. Studies that reported sample size were excluded from the pooled analysis if they were found to include data from convenient or pre-specified patient populations, or populations that overlapped with other included studies (e.g., publications from the same author/institution or registry/survey during a similar time frame). When studies overlapped, the publication with the largest reported patient population was retained.

### Study quality assessment

The research quality of relevant case series and cohort studies identified in the searches (characteristic publications only) was critically appraised using the Joanna Briggs Institute Critical Appraisal Tool and the Newcastle–Ottawa scale, respectively (Supplementary Table 3). Congress abstracts were not assessed for quality. Studies that received a poor assessment on the risk of bias appraisal were excluded.

## Results

### Literature search/screening

Among 1170 total publications identified in the initial literature searches (Embase, *n* = 642; MEDLINE, *n* = 417; Cochrane, *n* = 111), 193 publications met the final eligibility criteria, including 185 full-text publications and 8 congress abstracts (Fig. 1). A total of 155 publications provided information only on sample sizes for women and men with ATTR-CM, and 38 provided a description of ATTR-CM characteristics in patients of both sexes, as well as diagnostic techniques. Among the publications reporting sample sizes, 8 (5%) were based on controlled studies (randomized, *n* = 5; non-randomized, *n* = 3), and 147 (95%) were based on observational studies (retrospective, *n* = 87; prospective, *n* = 60). The ATTR-CM subtype breakdown in these studies was wild-type ATTR-CM, 58 (36%); hereditary ATTR-CM, 62 (40%); and undefined ATTR-CM, 67 (43%). (Some studies included multiple ATTR-CM subtypes.) Among the publications reporting disease characteristics, 30 (79%) were based on case series and 8 (21%) were based on cohort studies. Of these, the ATTR-CM subtype breakdown was wild-type ATTR-CM, 15 (39%); hereditary ATTR-CM, 29 (76%); and undefined ATTR-CM, 5 (13%).

Findings from 69 selected studies (sample size studies, *n* = 52; disease characteristics studies, *n* = 17) were pooled to assess ATTR-CM epidemiology. Twenty-five of the pooled studies (36%) included patients with wild-type ATTR-CM, 25 (36%) included patients with hereditary ATTR-CM, and 30 (43%) included patients with undefined ATTR-CM. (As above, some studies included multiple ATTR-CM subtypes.) Studies of wild-type, hereditary, and undefined ATTR-CM included 2027, 1279, and 1363 individuals, respectively.

### Frequency of wild-type and hereditary ATTR-CM

**Fig. 2:**
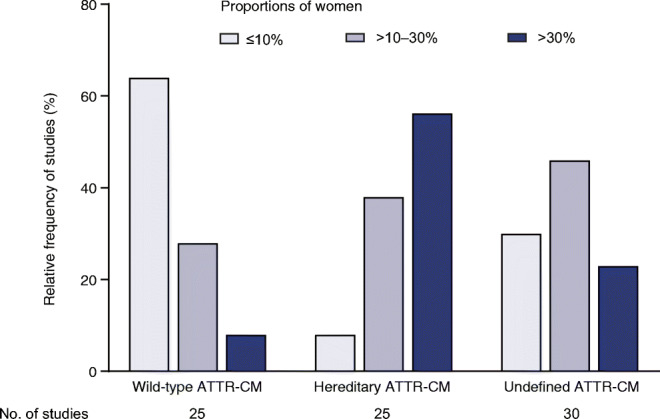
Relative frequency of studies in the pooled analysis that reported women by percentage range (*n* = 69). (Some studies included multiple ATTR-CM subtypes.) *ATTR-CM* transthyretin amyloid cardiomyopathy

Across studies included in the pooled analysis, 791 of 4669 (17%) patients with ATTR-CM were women. In the studies of wild-type, hereditary, and undefined ATTR-CM, 174 (9%), 366 (29%), and 251 (18%) patients were women, respectively. When the proportions of women were analyzed in ranges (i.e., ≤ 10%, > 10–30%, and > 30%), some differences between studies were evident (Fig. 2). For studies of wild-type and undefined ATTR-CM, the majority—92% (23/25) and 77% (23/30), respectively—reported that *less* than 30% of patients were women. In contrast, for studies of hereditary ATTR-CM, the majority—56% (14/25)—reported that *more* than 30% of patients were women.

### Sex-related differences in demographic and clinical characteristics

**Fig. 3:**
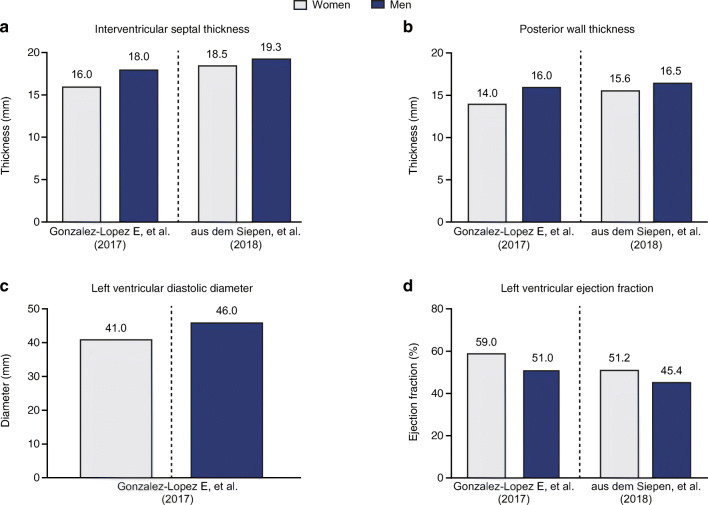
Echocardiographic measurements for women versus men with wild-type ATTR-CM in cohort studies. **a** Interventricular septal thickness. **b** Posterior wall thickness. **c** LV diastolic diameter. **d** LV ejection fraction [10, 31]. *ATTR-CM* transthyretin amyloid cardiomyopathy, *LV* left ventricular

Overall, no marked numerical differences between women and men with ATTR-CM in age or ethnicity were seen across studies that reported these characteristics (Table 1; Supplementary Tables 4–6) [24–40]. However, some anatomical differences in cardiac structure were identified **(**Fig. 3a–c); namely, women had numerically lower values for interventricular septal thickness, posterior wall thickness, and LV diastolic diameter compared with men in studies of wild-type [10, 31, 39], hereditary [29, 41–43], and undefined ATTR-CM [24, 26]. In addition, LV ejection fraction was numerically higher in women than men in several studies across ATTR-CM genotypes (Fig. 3d) [10, 31, 41, 44]. In studies that reported on survival and mortality [6, 10, 24, 35, 37, 38, 40, 41, 45–47], these outcomes did not appear to be different between women and men.

**Table 1:** Demographic and clinical characteristics by genotype, study type, and patient sex

**Characteristic**	**Wild-type ATTR-CM**^**a**^						**Hereditary ATTR-CM**^**b**^					
	**Case series (*n*** **= 7)**			**Cohorts (*n*** **= 5)**			**Case series (*n*** **= 22)**			**Cohorts (*n*** **= 4)**		
	**Studies** ***n*** **(%)**	**Men**	**Women**	**Studies** ***n*** **(%)**	**Men**	**Women**	**Studies** ***n*** **(%)**	**Men**	**Women**	**Studies** ***n*** **(%)**	**Men**	**Women**
Sample size, *n*	7 (100)	NR–9	1–5	5 (100)	5–668	1–43	22 (100)	1–45	1–21	4 (100)	37–227	12–96
Sample size, %	7 (100)	NR–89	11–100	5 (100)	82–93	6–18.5	22 (100)	1–91	9–100	4 (100)	67–80	20–33
Age, years												
Baseline, range	7 (100)	66–98	60–108	1 (20)	70–92	79	15 (68)	35–93	42–91	1 (25)	31–79	30–86
Onset, range/mean (SD)	0	NR	NR	1 (20)	76.1 (7.9)	82.3* (6.6)	9 (41)	22–80	24–78	0	NR	NR
Diagnosis, mean (SD)	0	NR	NR	1 (20)	77.4 (7.8)	83.9* (6.2)	0	NR	NR	1 (25)	49.6	47.4
TTR genotype, %												
Val30Met	NR						11 (50)	9–100	33–100	2 (50)	32–100	20–100
Val122Ile	–						4 (18)	7–75	50–100	1 (25)	71	29
Region, *n* (%)												
Europe	2 (29)	NR		4 (80)	NR		13 (59)	NR		4 (100)	NR	
North America	4 (57)	–		0	–		2 (9)	–		–	–	
Asia	1 (14)	–		1 (20)	–		8 (36)	–		–	–	
Multinational	–	–		–	–		1 (5)	–		–	–	
Race/ethnicity, %												
White	1 (14)	67	100	0	NR	NR	3 (14)	45–100	50–100	0	NR	
Black	2 (29)	33–100	0–100	0	NR	NR	4 (18)	33–100	50–100	0	NR	
Asian	1 (14)	100	100	1 (20)	100	100	8 (36)	100	100	0	NR	
Carpal tunnel syndrome, %	2 (29)	NR	50–100	2 (40)	35–49	25–43	5 (23)	11–100	0–100	0	NR	
NT-proBNP, pg/mL												
Range	2 (29)	51–458	431–3248	1 (20)	1576–8357	1720–11,171	7 (32)	134–14,056	220–9646	0	NR	
Mean (SD)	0	NR	NR	1 (20)	3514 (4988)	4232 (7509)	–	–	–	–	–	
Aortic stenosis, %	2 (29)	100	40–100	1 (20)	3	0	1 (5)	NR	100	0	NR	
Interventricular septal thickness, mm												
Range	1	(13–16)	(8–11)	2 (40)	18–19	16–19	10 (45)	11–27	7–26	1 (25)	10–20	10–16
Mean (SD)	–	–	–	–	–	–	1 (5)	NR	16 (1)	1 (25)	–	11.6 (2.9)
Posterior wall thickness, mm												
Range	1	(13–16)	(12–15)	2 (40)	16–16.5	14–15.6	6 (23)	11–25	10–24	1 (25)	10–13	9–10
Mean (SD)	–	–	–	–	–	–	–	–	–	1 (25)	13.3 (3.6)	11.4 (2.5)
LV diastolic diameter, mm	0	NR		1 (20)	46	41	3 (14)	42–54	31–54	1 (25)	42–52	39–50
LV mass, g												
Mean (SD)	0	NR		1 (20)	513 (162)	281 (1)	0	NR		–	90–209	81–152
Ejection fraction, %												
Range	4 (57)	50–80	57–77	2 (40)	45–51	51–59	5 (23)	25–73	30–61	1 (25)	58–71	57–74
Mean (SD)	–	–	–	–	–	–	1 (5)	NR	50 (1)	1 (25)	58.6 (10.2)	66 (12.4)
Atrial fibrillation, %	2 (33)	25–33	33–40	2 (40)	56–61	47–55	2 (9)	7–33	33–100	0	NR	
**Characteristic**	**Studies of undefined ATTR-CM**^**c**^											
	**Case series (*n*** **= 3)**					**Cohorts (*n*** **= 1)**						
	**Studies** ***n*** **(%)**		**Men**		**Women**	**Studies** ***n*** **(%)**			**Men**		**Women**	
Sample size, *n*	3 (100)		1–15		2–10	1 (100)			45		6	
Sample size, %	3 (100)		33–60		40–67	1 (100)			88		12	
Age, years												
Baseline, range	3 (100)		59–97		64–91	0			NR			
Region, *n* (%)												
Europe	1 (33)		–		–	1 (100)			–			
North America	2 (67)		–		–	0			NR			
Race/ethnicity, %												
Black	1 (33)		100		100	0			NR			
Carpal tunnel syndrome, %	1 (33)		38		0	0			NR			
NT-proBNP, pg/mL												
Range	1 (33)		(124–7846)		(959–2799)	0			NR			
Aortic stenosis, %	1 (33)		100		50	0			NR			
Interventricular septal thickness, mm												
Range	1 (33)		22^d^		(13–14)	0			NR			
Mean (SD)	1 (33)		16 (1.5)		17.25 (2.1)	0			NR			
Posterior wall thickness, mm												
Mean (SD)	1 (33)		16 (1.7)		16.5 (1.7)	0			NR			
LV diastolic diameter, mm												
Mean (SD)	1 (33)		53.5 (8.8)		46 (9.5)	0			NR			
LV mass, g	0		NR			1 (100)			106–140	95–116		
Ejection fraction, %												
Range	1 (33)		55^d^		56–69	0			NR	NR		
Mean (SD)	0		NR		NR	1 (100)			55 (12)	62 (14)		
Atrial fibrillation, %	2 (67)		0–7		10–33	0			NR			

*ATTR-CM* transthyretin amyloid cardiomyopathy, *LV* left ventricular, *NR* not reported, *NT-proBNP* N-terminal pro-B-type natriuretic peptide, *SD* standard deviation

*P < 0.01

^a^Three studies were excluded because they did not include data for women with wild-type ATTR-CM

^b^Three studies were excluded because they included only women or only men with hereditary ATTR-CM

^c^One study was excluded because it did not include data for women with ATTR-CM

^d^Based on limited data (*n* = 1)

### Screening/diagnostic techniques in ATTR-CM

**Fig. 4:**
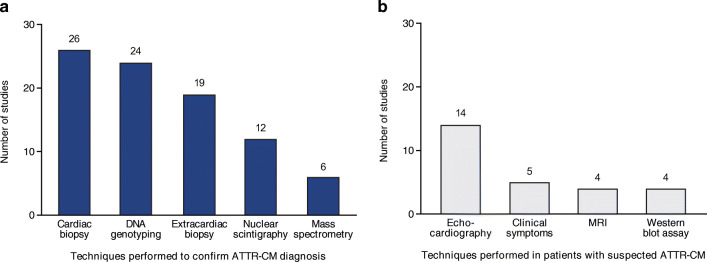
Techniques used **a** to confirm a diagnosis of ATTR-CM or **b** to investigate suspected ATTR-CM (*n* = 38). *ATTR-CM* transthyretin amyloid cardiomyopathy, *MRI* magnetic resonance imaging

A number of tests may be conducted in patients with suspected ATTR-CM to examine the risk that the condition is present. In the studies included in this review, echocardiography was the most frequently reported test, conducted in 14 of 38 (37%) studies [6, 10, 26, 31–33, 37, 39, 41, 42, 44, 48–50] (Fig. 4). Clinical symptoms (5 [13%]), magnetic resonance imaging (MRI; 4 [11%]), and Western blot assay (4 [11%]) were other features or techniques used to screen for ATTR-CM in similar proportions of studies.

Multiple approaches can be taken to establish a diagnosis of ATTR-CM, including endomyocardial or extracardiac biopsy, mass spectrometry, and nuclear scintigraphy; in addition, DNA genotyping can be used to confirm the presence or absence of a *TTR* gene mutation after the diagnosis is established [18]. The number of published studies including these diagnostic techniques, alone or in combination, is shown in Fig. 4. The most common were cardiac biopsy [6, 10, 24–26, 28, 31–39, 41, 43, 44, 46, 47, 49–54], DNA genotyping [6, 10, 25, 27–30, 33, 37, 40–45, 47, 48, 51–53, 55–58], and extracardiac biopsy, performed in 26 (68%), 24 (63%), and 19 (50%) studies, respectively. Biopsy was not performed in 6 (16%) studies [27, 29, 30, 40, 55, 57], but DNA genotyping was conducted in all 6 of these studies and nuclear scintigraphy in 2 of the 6 studies. Cardiac and extracardiac biopsies were conducted together in 14 (37%) [6, 10, 26, 28, 33, 39, 41, 44, 46, 51–54] studies, and cardiac biopsy and DNA genotyping were conducted together in 13 (34%) studies [6, 10, 25, 28, 33, 37, 41, 43, 44, 47, 51–53].

## Discussion

Based on a pooled analysis of 69 studies, among 4669 patients with ATTR-CM, we found that 17% were women and 83% were men. The lowest proportion of women (9%) was observed in studies of wild-type ATTR-CM, whereas the highest (29%) was observed in studies of hereditary ATTR-CM. These findings are consistent with previous reports that ATTR-CM is a condition predominantly identified in men [6, 39], especially for the wild-type versus the hereditary subtype [6–8].

The research reviewed in the current publication does not elucidate the potential underlying mechanisms for the predominance of this condition in men or the higher proportion of women presenting with hereditary versus wild-type ATTR-CM, nor does it capture potential bias in reporting. Sex hormones and sex chromosomes have been shown to account for sex differences in cardiovascular disease [59, 60], and findings from several studies suggest that these factors may also help explain discrepancies in the prevalence and onset of ATTR-CM between men and women [41, 54, 61–63]. In a study of differences in penetrance of hereditary amyloidosis (i.e., the Val30Met ATTR pathogenic variant), Hellman et al. observed that penetrance of the trait was significantly higher when inherited from the mother than from the father [62]. In an earlier Swedish genealogical study, the age of onset was shown to be significantly higher when the mutation was inherited from the father, and anticipation (i.e., higher penetrance in younger generations) was greater in descendants of mothers with the disease [63]. It is also possible that genetic counseling and mutations identified within families with a history of hereditary disease improve detection of this genotype in women, particularly as women may be more inclined to seek the help of healthcare providers [64–66]. These sex-biasing factors are complex, and many other factors, including age and ascertainment bias, are also expected to play a role.

The large degree of heterogeneity between studies reporting characteristics of ATTR-CM prevented meta-analysis of the data, but we observed trends suggestive of potential sex-based differences. Across studies, lower interventricular septal and posterior wall thickness and higher LV ejection fractions were reported in women compared with men. These trends are consistent with reported measurements for normal and abnormal hearts [67]. A suggested cut-off criterion for the diagnosis of ATTR-CM is an LV wall thickness > 12 mm in both sexes [23]. Given that both normal and abnormal cardiac anatomy is smaller in women than men, which our findings suggest is also the case for women and men with ATTR-CM, women with ATTR-CM could be at risk for potential underdiagnosis because they are less likely to meet the diagnostic wall-thickness threshold. Further research is warranted to determine whether use of lower cut-off values or cardiac dimensions indexed by body size for women will affect rates of suspicion and ultimately diagnosis of ATTR-CM in women.

Various approaches may be used to help raise suspicion of ATTR-CM and facilitate earlier identification of the disease, including echocardiogram (ECHO), electrocardiogram (ECG), cardiac magnetic resonance imaging (cMRI), and biomarker clues [18, 23, 68]. ECHO was the most frequently reported test conducted in the studies in this review, reflecting the prominent role this test has traditionally played in the evaluation of cardiac structure and function in patients with troubling heart symptoms. However, the limited sensitivity and specificity of ECHO for ATTR-CM may contribute to missed or late diagnoses of amyloidosis. Although ECG may be useful in identifying ATTR-CM, none of the studies reported use of this diagnostic technique. Similarly, no studies used the cardiac biomarkers N-terminal pro-B-type natriuretic peptide or troponin to confirm the diagnosis of ATTR-CM, although these markers reportedly predict mortality in patients with wild-type ATTR-CM and are thus used for risk stratification [69]. Therefore, our findings suggest that additional guidance on the assessment of patients with suspected ATTR-CM is needed to help raise awareness among clinicians and encourage earlier diagnosis.

In recently published multi-societal expert consensus recommendations, several diagnostic criteria were proposed for ATTR-CM, primarily based on endomyocardial biopsy, extracardiac biopsy, or scintigraphy, as were guidelines for the appropriate use of multimodality imaging [18]. The majority of studies included in the current systematic review were designed and conducted prior to publication of these recommendations/guidelines (in 2019), but the study findings related to diagnostic testing are nonetheless of interest. The use of non-invasive scintigraphy was not commonly reported in this SLR, but substantial research supports its utilization. In a large, multicenter study in > 1000 patients with biopsy-proven cardiac amyloidosis, Gillmore et al. demonstrated that positive nuclear cardiac scintigraphy was 100% specific for ATTR-CM when light-chain amyloidosis (AL) was concurrently ruled out in patients with a visual myocardial uptake grading of 2 and 3 [19]. The range of diagnostic tests reported in the studies included in our review (i.e., cardiac and extracardiac biopsy, mass spectrometry, DNA genotyping, and scintigraphy) does not reveal a breach between the consensus ATTR-CM diagnostic recommendations and practice in clinical research. However, we found some evidence of inconsistencies in the approach: for example, cardiac and extracardiac biopsies were conducted together in 14 (37%) studies, and cardiac biopsy and DNA genotyping were conducted together in 13 (34%) studies. These findings indicate that broader education on the consensus recommendations is needed to encourage more uniform application of assessment and diagnostic criteria.

A number of limitations should be considered when assessing the findings of this SLR, including the small sample sizes of the studies, particularly for women, and the limited data available, particularly in cohort studies. The majority of these data were derived from case series rather than cohort studies, impeding direct comparison of data between women and men. Other limitations include heterogeneity in the stage at which patients were recruited in the studies (i.e., previously diagnosed patients, patients with suspected ATTR-CM, and patients pre-selected for another condition), heterogeneity in the statistical reporting of data (e.g., means vs. medians), and heterogeneity in time frames and duration of follow-up (i.e., from onset or time of biopsy or pre- and post-liver transplant to variable follow-up visit or death). Finally, the criteria used to confirm the presence of ATTR-CM (e.g., interventricular septal thickness > 12 mm, positive biopsy or scintigraphy) may have biased patient selection and may have been inaccurate for identifying women with ATTR-CM.

The results of this literature review indicate that ATTR-CM is a condition predominantly reported in men compared with women. The disparity between sexes in ATTR-CM frequency may be explained by several factors, including potential cardioprotective effects of estrogen, smaller heart structures in women that do not meet diagnostic thresholds for ATTR-CM, and a lack of awareness of red flags for ATTR-CM in women compared with men. Interpretation of the literature was limited by the heterogeneous nature of the studies included and the small patient populations involved, but our findings raise important questions and reinforce the need for additional investigation into sex-related differences in ATTR-CM.

## Electronic supplementary material

ESM 1 (DOCX 171 kb)
