# Supplementary material for: Transthyretin amyloid cardiomyopathy in women: frequency, characteristics, and diagnostic challenges
Source: Heart Fail Rev. 2020 Aug 14;26(1):35–45. doi: 10.1007/s10741-020-10010-8 (PMC7769788; doi:10.1007/s10741-020-10010-8)
Supplement: Supplementary file 1 — (DOCX 171 kb) [file 10741_2020_10010_MOESM1_ESM.docx]

# Supplementary Material

**Transthyretin amyloid cardiomyopathy in women: frequency, characteristics, and diagnostic challenges**

***Authors:*** Marianna Bruno^1^, Adam Castaño^1^, Arianna Burton^1^, Justin L. Grodin^2^

^1^Medical Affairs, Pfizer Inc, New York, NY; ^2^Department of Internal Medicine, University of Texas Southwestern Medical Center, Dallas, TX

**Supplementary Table 1.** Search strategy used for MEDLINE, Embase, and Cochrane databases.

| Step | Search string |
| --- | --- |
| **1** | (transthyretin or (prealbumin adj4 amyloid*) or amyloid transthyretin or familial amyloid* or hereditary amyloid* or ATTR or TTR or transthyretin amyloid* or transthyretin-related or transthyretin-associated or transthyretin-mediated or transthyretin-type or wtATTR or mATTR or hATTR or vATTR or ATTRwt or ATTRm or ATTRv).ti,ab. |
| **2** | (cardiac or cardiomyopathy or heart).ti,ab. |
| **3** | 1 and 2 |
| **4** | (ATTR-CM or ATTR CM or ATTR-CA or ATTR CA).ti,ab. |
| **5** | (cardiac amyloid* or senile amyloid* or amyloid cardiomyopathy).ti,ab. |
| **6** | 3 or 4 or 5 |
| **7** | (Female* or woman or women or gender or sex).mp. |
| **8** | (epidemiol* or prevalen* or inciden* or mortality).ti,ab. |
| **9** | 6 and (7 or 8) |
| **10** | randomized controlled trial.pt. |
| **11** | controlled clinical trial.pt. |
| **12** | systematic review.pt. |
| **13** | (systematic review$ or meta?analys$ or network meta?analys$ or NMA).ti,ab. |
| **14** | randomi?ed.ab. |
| **15** | placebo.tw. |
| **16** | drug therapy.fs. |
| **17** | clinical trials as topic.sh. |
| **18** | randomly.ab. |
| **19** | trial.ab. |
| **20** | groups.ab. |
| **21** | (crossover or cross-over or cross over).tw. |
| **22** | ((singl$ or double$ or triple$ or treble$) and (blind$ or mask$)).tw,sh. |
| **23** | (case control or case-control).ti,ab. |
| **24** | ((follow up or follow-up) adj (study or studies)).ti,ab. |
| **25** | (Longitudinal or retrospective or prospective or comparative or cohort or cross sectional or cross-sectional).ti,ab. |
| **26** | ((observ$ or registry) adj3 (study or studies)).ti,ab. |
| **27** | or/10-26 |
| **28** | 9 and 27 |
| **29** | (animals not (humans and animals)).sh. |
| **30** | 28 not 29 |
| **31** | (conference abstract or comment* or letter or note or editorial or case report or case series or case study or erratum).pt. |
| **32** | 30 not 31 |
| **33** | limit 32 to English language |
| **34** | remove duplicates from 33 |

**Supplementary Table 2.** ATTR-CM diagnostic criteria (based on the recommendations of the 2019 Multi-societal Expert Consensus Committee) [1].

|  |  | |
| --- | --- | --- |
| 1 | **Histological diagnosis of ATTR-CM: Endomyocardial biopsy** | Endomyocardial biopsy positive for cardiac amyloidosis with Congo red staining; typing by immunohistochemistry and/or mass spectrometry |
| OR 2 | **Histological diagnosis of ATTR-CM: Extracardiac biopsy**  **AND** | Extracardiac biopsy-proven ATTR amyloidosis |
|  |  | Typical cardiac imaging features (usually IVST or LVWT >12 mm) |
| OR 3 | **Clinical diagnosis of ATTR-CM:**  **^99m^Tc-PYP, ^99m^Tc***-***DPD, ^99m^Tc***-***HMDP**  **AND**  **AND** | ^99m^Tc-PYP, ^99m^Tc-DPD, ^99m^Tc*-*HMDP Grade 2 or 3 myocardial uptake of radiotracer |
|  |  | Absence of a clonal plasma cell process |
|  |  | Typical cardiac imaging features (e.g. IVST or LVWT >12 mm) |

*ATTR*, transthyretin amyloidosis; *ATTR-CM*, transthyretin amyloid cardiomyopathy; *IVST*, interventricular septal thickness; *LVWT*, left ventricular wall thickness; *^99m^Tc-DPD*, technetium-99m 3,3-diphosphono-1,2-propanodicarboxylic acid; *^99m^Tc-HMDP*, technetium-99m hydroxymethylene diphosphonate; *^99m^Tc-PYP* technetium-99 mpyrophosphate

**Supplementary Table 3.** Quality assessment tools used for critical appraisal of cohort studies and case series identified in searches.

| **Joanna Briggs Institute Critical Appraisal Tool  (Case series)** | **Newcastle–Ottawa Quality Assessment Scale  (Cohort studies)** |  |  |
| --- | --- | --- | --- |
| 1. Were there clear criteria for inclusion in the case series? 2. Yes 3. No/Unclear 4. Was the condition measured in a standard, reliable way for all participants included in the case series? 5. Yes 6. No/Unclear 7. Were valid methods used for identification of the condition for all participants included in the case series? 8. Yes 9. No/Unclear 10. Did the case series have consecutive inclusion of participants? 11. Yes 12. No/Unclear 13. Did the case series have complete inclusion of participants? 14. Yes 15. No/Unclear 16. Was there clear reporting of the demographics of the participants in the study? 17. Yes 18. No/Unclear 19. Was there clear reporting of clinical information of the participants? 20. Yes 21. No/Unclear 22. Were the outcomes or follow-up results of cases clearly reported? 23. Yes 24. No/Unclear 25. Was there clear reporting of the presenting site(s)/clinic(s) demographic information? 26. Yes 27. No/Unclear 28. Was statistical analysis appropriate? 29. Yes 30. No/Unclear | **Selection**   1. Representativeness of the exposed cohort:   a) truly representative of the average _______________ (describe) in the community*  b) somewhat representative of the average ______________ in the community*  c) selected group of users (e.g. nurses, volunteers)  d) no description of the derivation of the cohort   1. Selection of the non-exposed cohort:   a) drawn from the same community as the exposed cohort*  b) drawn from a different source  c) no description of the derivation of the non-exposed cohort   1. Ascertainment of exposure:   a) secure record (e.g. surgical records)*  b) structured interview*  c) written self-report  d) no description   1. Demonstration that outcome of interest was not present at start of study   a) Yes*  b) No  **Comparability**   1. Comparability of cohorts on the basis of the design or analysis:   a) study controls for _____________ (select the most important factor)*  b) study controls for any additional factor* (This criterion could be modified to indicate specific control for a second important factor.)  **Outcome**   1. Assessment of outcome:   a) independent blind assessment*  b) record linkage*  c) self-report  d) no description   1. Was follow-up long enough for outcomes to occur?   a) Yes (select an adequate follow-up period for outcome of interest)*  b) No   1. Adequacy of follow-up of cohorts:   a) complete follow-up: all subjects accounted for*  b) subjects lost to follow-up unlikely to introduce bias: small number lost > ____ % (select an adequate %) follow-up, or description provided of those lost) ___  c) follow-up rate < ____% (select an adequate %) and no description of those lost  d) no statement |  |  |

**Supplementary Table 4.** Demographic and clinical characteristics of patients with wild-type ATTR-CM by patient sex in **(A)** case series and **(B)** cohort studies.

**A.** Case series (*n* = 9^a^)

| **Characteristic** | **Helder 2014 [2]** | | **Satoskar 2011 [3]** | | **Sharma 2008 [4]** | | **Treibel 2016 [5]** | | **Xu 2019 [6]** | | **Yamamoto 2012 [7]** | | **Zegri-Reiriz 2019 [8]** | |
| --- | --- | --- | --- | --- | --- | --- | --- | --- | --- | --- | --- | --- | --- | --- |
|  | **Men** | **Women** | **Men** | **Women** | **Men** | **Women** | **Men** | **Women** | **Men** | **Women** | **Men** | **Women** | **Men** | **Women** |
| **Sample size, *n* (%)** | 8 (89) | 1^b^ (11) | 3 (75) | 1^b^ (25) | 4 (57) | 3 (43) | 4 (67) | 2 (33) | 9 (60) | 5 (40) | 6 (75) | 2 (25) | 0 (0) | 2 (100) |
| **Age, years**  **Range** | 66–81 | 77 | 66–80 | 68 | 71–98 | 60–108 | 69–85 | 73–80 | 72–89 | 76–85 | 78–90 | 83–90 | — | 85–86 |
| **Country** | USA | | USA | | USA | | UK | | USA | | Japan | | Spain | |
| **Race/Ethnicity, %**  **White**  **Black**  **Asian** | NR | | 67  33  0 | 100  0  0 | 0  100  0 | 0  100  0 | NR | | NR | | 0  0  100 | 0  0  100 | NR | |
| **Carpal tunnel syndrome, *n* (%)** | NR | | NR | | NR | | 0 (0) | 1 (50) | NR | | NR | | — | 2 (100) |
| **NT-proBNP, pg/mL**  **Range** | NR | | NR | | NR | | 51–458 | 431–510 | NR | | NR | | — | 842–3248 |
| **Aortic stenosis, *n* (%)** | NR | | NR | | NR | | 4 (100) | 2 (100) | 3 (33) | 2 (40) | NR | | NR | |
| **Interventricular septal thickness, mm**  **Range** | NR | | NR | | NR | | NR | | NR | | 13–16 | 8–11 | NR | |
| **Posterior wall thickness, mm**  **Range** | NR | | NR | | NR | | NR | | NR | | 13–16 | 12–15 | NR | |
| **LV diastolic diameter, mm** | NR | | NR | | NR | | NR | | NR | | NR | | NR | |
| **LV mass index, g/m**  **Range** | NR | | NR | | NR | | 93–150 | 117–137 | NR | | NR | | NR | |
| **Ejection fraction, %**  **Range** | 60–80 | 76 | NR | | NR | | 64–77 | 61–77 | NR | | 50–66 | 58–60 | — | 57–61 |
| **Atrial fibrillation, *n* (%)** | NR | | NR | | 1 (25) | 1 (33) | NR | | 3 (30) | 2 (40) | NR | | NR | |

^a^Two studies are not included in this table as they did not report data for women in this category.

^b^Absolute values are reported in rows below for the single individual in this subgroup.

*ATTR-CM*, transthyretin amyloid cardiomyopathy*; LV*, left ventricular; *NR*, not reported; *NT-proBNP*, N-terminal pro-B-type natriuretic peptide

**B.** Cohort studies (*n* = 6^a^)

| **Characteristic** | **aus dem Siepen 2018 [9]** | | | **Gonzalez Lopes 2017 [10]** | | **Kim 2019 [11]** | | **Lane 2019 [12]** | | **Rapezzi 2009 [13]** | |
| --- | --- | --- | --- | --- | --- | --- | --- | --- | --- | --- | --- |
|  | **Men** | | **Women** | **Men** | **Women** | **Men** | **Women** | **Men** | **Women** | **Men** | **Women** |
| **Sample size, *n* (%)** | 176 (92) | | 15 (8) | 88 (81) | 20 (19) | 5 (83) | 1^b^ (17) | 668 (94) | 43 (6) | 14 (93) | 1 (7) |
| **Age, years**  **Range**  **Mean (SD)** | —  73.6 (6.0) | | —  75.1 (8.6) | NR | NR | 70–92  — | 79  —  — | NR | NR | NR | NR |
| **Country** | Germany | | | Italy/Spain | | South Korea | | UK | | Italy | |
| **Race/Ethnicity** | NR | | | NR | | NR | | NR | | NR | |
| **Carpal tunnel syndrome, *n* (%)** | 81 (49) [*N* = 166] | 6 (43) [*N* = 14] | | 31 (35) | 5 (25) | NR | | NR | | NR | |
| **NT-proBNP, pg/mL  Median (IQR)   Mean (SD)** | —  3514 (4988) [*N* = 171] | —  4232 (7509) [*N* = 14] | | 2942  (1576–8357)  — | 6177* (1720–11,712)  — | NR | | NR | | NR | |
| **Aortic stenosis, *n* (%)** | NR | NR | | 1 (3) | 0 (0) | NR | | NR | | NR | |
| **Interventricular septal thickness, mm**  **Mean (SD)** | 19.3 (3.7) | 18.5 (4.1) | | 18 (3) | 16* (3) | NR | | NR | | NR | |
| **Posterior wall thickness, mm  Mean (SD)** | 16.5 (3.2) | 15.6 (2.8) | | 16 (3) | 14* (3) | NR | | NR | | NR | |
| **LV diastolic diameter, mm**  **Mean (SD)** | NR | | | 46 (6) | 41* (6) | NR | | NR | | NR | |
| **LV mass index, g/m**  **Mean (SD)** | 234 (68) | 224 (65) | | 212 (69) | 158* (52) | NR | | NR | | NR | |
| **Ejection fraction, %**  **Mean (SD)** | 45.4 (16.3) | 51.2 (14.9) | | 51 (13) | 59* (13) | NR | | NR | | NR | |
| **Atrial fibrillation, *n* (%)** | 102 (61) | 7 (47) | | 49 (56) | 11 (55) | NR | | NR | | NR | |

**P* < 0.05, for comparison of women vs. men.

^a^One study is not included in this table as it did not report data for women in this category.

^b^Absolute values are reported in the rows below for the single individual in this subgroup.

*ATTR-CM*, transthyretin amyloid cardiomyopathy*; IQR, interquartile range;* *LV*, left ventricular; *NR*, not reported; *NT-proBNP*, N-terminal pro-B-type natriuretic peptide; *SD*, standard deviation

**Supplementary Table 5.** Demographic and clinical characteristics of patients with hereditary ATTR-CM by patient sex in **(A)** case series and **(B)** cohort studies.

**A.** Case series (*n* = 26^a^)

| **Characteristic** | **Abulizi 2018 [14]** | | | **Choi 2018 [15]** | | **Damy 2016 [16]** | | | **Di Bella 2010 [17]** | | **Galat 2016 [18]** | | | **Gustafsson 2012 [19]** | | |
| --- | --- | --- | --- | --- | --- | --- | --- | --- | --- | --- | --- | --- | --- | --- | --- | --- |
|  | **Men** | | **Women** | **Men** | **Women** | **Men** | **Women** | | **Men** | **Women** | **Men** | | **Women** | **Men** | | **Women** |
| **Sample size, *n* (%)** | 4 (67) | | 2 (33) | 6 (67) | 3 (33) | 11 (73) | 4 (27) | | 5 (83) | 1^b^ (17) | 0 | | 1^b^ (100) | 7 (70) | | 3 (30) |
| **ATTR fibril composition, *n* (%)** | NR | | | NR | | NR | | | NR | | NR | | | Type A: 7 (64)  Type B: 4 (36) | | Type A: 3 (100)  Type B: 1^b^ (100) |
| ***TTR* genotype, *n* (%)**  **Val30Met**  **Val122Ile**  **Other** | 0 (0)  3 (75)  1 (25) | | 0 (0)  1 (50)  1 (50) | 0 (0)  0 (0)  6 (100) | 0 (0)  0 (0)  3 (100) | 0 (0)  0 (0)  11 (100) | 0 (0)  0 (0)  4 (100) | | 0 (0)  0 (0)  5 (100) | 0 (0)  0 (0)  1 (100) | —  —  — | | 1 (100)  0 (0)  0 (0) | 7 (100)  0 (0)  0 (0) | | 1 (33.3)  0 (0)  2 (66.6) |
| **Age, years**  **Range** | 66–77 | | 68–81 | NR | | NR | | | 35–78 | 45 | — | | 78 | Type A: 55–63  Type B: 62–67 | | Type A: 42–63  Type B: 57 |
| **Country** | France | | | South Korea | | France | | | Italy | | France | | | Sweden | | |
| **Race/Ethnicity, %**  **White**  **Black**  **Asian** | NR | | | NR | | 5 (45)  6 (55)  0 (0) | 2 (50)  2 (50)  0 (0) | | NR | | NR | | | NR | | |
| **Carpal tunnel syndrome, *n* (%)** | NR | | | 2 (33.3) | 1 (33.3) | 4 (36) | 2 (50) | | NR | | — | | 0 (0) | NR | | |
| **NT-proBNP, pg/mL**  **Range** | 2190–3673 | | 1396–6362 | NR | | 506–10775 | 3290–9646 | | NR | | — | | 959 | *Pre-transplant:*  Type A: 117–494  Type B: NR  *Post-transplant:* Type A: 586–25,096  Type B: 4905–9575 | *Pre-transplant:* Type A: NR  Type B: NR  *Post-transplant:* Type A: 4383–7735  Type B: 4406 | |
| **Aortic stenosis, *n* (%)** | NR | | | NR | | NR | | | NR | | NR | | | NR | | |
| **Interventricular septal thickness, mm**  **Range** | 15–18 | | 22 | NR | | NR | | | NR | | — | | 16 | NR | | |
| **Posterior wall thickness, mm**  **Range** | NR | | | NR | | 16–25 | 15–20 | | NR | | NR | | | NR | | |
| **LV diastolic diameter, mm** | NR | | | NR | | NR | | | NR | | NR | | | NR | | |
| **LV mass index, g/m** | NR | | | NR | | NR | | | NR | | NR | | | NR | | |
| **Ejection fraction, %**  **Range** | 33–65 | 36–55 | | NR | | 30–60 | | 35–45 | NR | | — | 50 | | NR | | |
| **Atrial fibrillation, *n* (%)** | NR | | | NR | | NR | | | NR | | — | 1 (100) | | NR | | |

**Table 5A (*continued*)**

| **Characteristic** | **Haagsma 2007 [20]** | | | **Hattori 2003 [21]** | | | **Ihse 2013 [22]** | | **Jacobson 1997 [23]** | | **Kero 2020 [24]** | | **Koike 2011 [25]** | |
| --- | --- | --- | --- | --- | --- | --- | --- | --- | --- | --- | --- | --- | --- | --- |
|  | **Men** | **Women** | | **Men** | **Women** | | **Men** | **Women** | **Men** | **Women** | **Men** | **Women** | **Men** | **Women** |
| **Sample size, *n* (%)** | 3 (43) | 4 (57) | | 7 (47) | 8 (53) | | 45 (68) | 21 (32) | 3 (50) | 3 (50) | 1^b^ (50) | 1^b^ (50) | 10 (91) | 1^b^ (9) |
| **ATTR fibril composition, *n* (%)** | NR | | | NR | | | *Val30Met:*  Type A: 1 (25)  Type B: 3 (75)  *Non-Val30Met:*  Type A: 40 (98)  Type B: 1 (2) | *Val30Met:*  Type A: 0 (0)  Type B: 2 (100)  *Non-Val30Met:*  Type A: 18 (95)  Type B: 1 (53) | NR | | NR | | NR | |
| ***TTR* genotype, *n* (%)**  **Val30Met**  **Val122Ile**  **Other** | 2 (67)  0 (0)  1 (33) | | 0 (0)  0 (0)  4 (100) | 7 (100)  0 (0)  0 (0) | | 4 (50)  0 (0)  4 (50) | 4 (9)  3 (7)  38 (84) | 2 (10)  1 (5)  18 (86) | 0 (0)  3 (100)  0 (0) | 0 (0)  3 (100)  0 (0) | NR | | 10 (100)  0 (0)  0 (0) | 1 (100)  0 (0)  0 (0) |
| **Age, years**  **Range** | 53–58 | | 49–54 | NR | | | NR | | 71–93 | 84–91 | 63 | 67 | 54–76 | 64 |
| **Country, *n* (%)** | the Netherlands:  7 (100) | | | Japan  15 (100) | | | *Val30Met:*  Japan: 4 (100)  *Non-Val30Met:*  Italy: 23 (56)  USA: 7 (17)  Sweden: 6 (15)  Japan: 3 (7)  UK: 1 (2)  Ireland: 1 (2) | *Val30Met:* Japan: 2 (100)  *Non-Val30Met:*  Italy: 7 (37)  Japan: 5 (26)  Sweden: 4 (21)  USA: 3 (16) | USA  6 (100) | | Sweden  2 (100) | | Japan  11 (100) | |
| **Race/Ethnicity, *n* (%)**  **White**  **Black**  **Asian** | NR | | | 0 (0)  0 (0)  7 (100) | | 0 (0)  0 (0)  8 (100) | NR | | 0 (0)  3 (100)  0 (0) | 0 (0)  3 (100)  0 (0) | NR | | 0 (0)  0 (0)  10 (100) | 0 (0)  0 (0)  1 (100) |
| **Carpal tunnel syndrome, *n* (%)** | NR | | | NR | | | NR | | NR | | NR | | NR | |
| **NT-proBNP, pg/mL**  **Range** | 815–14,056 | | 862–2108 | NR | | | NR | | NR | | 962 | 1973 | NR | |
| **Aortic stenosis, *n* (%)** | NR | | | NR | | | NR | | NR | | NR | | NR | |
| **Interventricular septal thickness, mm**  **Range** | *Pre-transplant:* 11–13  *Post-transplant:* 19–21 | | *Pre-transplant:* 7–16  *Post-transplant:* 11–20 | NR | | | *Val30Met:*  Autopsy: 30  Alive: 11–27  *Non-Val30Met:* 13–27 | *Val30Met:* NR  *Non-Val30Met:* 10–22 | NR | | NR | | 11–18 | 16 |
| **Posterior wall thickness, mm**  **Range** | NR | | | NR | | | *Val30Met:*  Autopsy: 18–25  Alive: 12–15  *Non-Val30Met:* 12–22 | *Val30Met:*  Autopsy: 13–17  Alive: —  *Non-Val30Met:* 10–22 | NR | | NR | | NR | |
| **LV diastolic diameter, mm** | NR | | | NR | | | NR | | NR | | NR | | NR | |
| **LV mass index, g/m** | NR | | | NR | | | NR | | NR | | NR | | NR | |
| **Ejection fraction, %** | NR | | | NR | | | NR | | NR | | NR | | NR | |
| **Atrial fibrillation, *n* (%)** | NR | | | NR | | | NR | | 1 (33) | 1 (33) | NR | | NR | |

**Table 5A (*continued*)**

| **Characteristic** | **Morner 2005 [26]** | | | **Nelson 2013 [27]** | | | **Nelson 2015 [28]** | | **Okamoto 2008 [29]** | | **Oshima 2014 [30]** | | **Pilebro 2018 [31]** | |
| --- | --- | --- | --- | --- | --- | --- | --- | --- | --- | --- | --- | --- | --- | --- |
|  | **Men** | | **Women** | **Men** | | **Women** | **Men** | **Women** | **Men** | **Women** | **Men** | **Women** | **Men** | **Women** |
| **Sample size, *n* (%)** | 2 (50) | | 2 (50) | 4 (57) | | 3 (43) | 4 (67) | 2 (33) | 5 (83) | 1^b^ (17) | 7 (64) | 4 (36) | 4 (67) | 2 (33) |
| **ATTR fibril composition, *n* (%)** | NR | | | NR | | | NR | | NR | | NR | | Type A: 2 (50)  Type B: 2 (50) | Type A: 2 (100)  Type B: 0 (0) |
| ***TTR* genotype, *n* (%)**  **Val30Met**  **Val122Ile**  **Other** | 2 (100)  0 (0)  0 (0) | | 2 (100)  0 (0)  0 (0) | 0 (0)  0 (0)  4 (100) | | 0 (0)  0 (0)  3 (100) | 0 (0)  0 (0)  4 (100) | 0 (0)  0 (0)  2 (100) | NR | | 7 (100)  0 (0)  0 (0) | 4 (100)  0 (0)  0 (0) | 4 (100)  0 (0)  0 (0) | 2 (100)  0 (0)  0 (0) |
| **Age, years**  **Range** | 68–77 | | 71–77 | 41–51 | | 46–53 | 39–54 | 44–49 | NR | | NR | | 66–69 | 68–76 |
| **Country** | Sweden | | | Denmark | | | Denmark | | Japan | | Japan | | Sweden | |
| **Race/Ethnicity, *n* (%)**  **White**  **Black**  **Asian** | NR | | | NR | | | NR | | 0 (0)  0 (0)  5 (100) | 0 (0)  0 (0)  1 (100) | 0 (0)  0 (0)  7 (100) | 0 (0)  0 (0)  4 (100) | NR | |
| **Carpal tunnel syndrome, *n* (%)** | NR | | | 2 (50) | 2 (67) | | NR | | NR | | NR | | NR | |
| **NT-proBNP, pg/mL**  **Range** | NR | | | NR | | | NR | | NR | | NR | | 134–3256 | 220–328 |
| **Aortic stenosis, *n* (%)** | NR | | | NR | | | NR | | NR | | NR | | NR | |
| **Interventricular septal thickness, mm**  **Range** | 21–23 | | 20–26 | 13–17 | 16–18 | | 12–17 | 7–10 | *Pre-transplant:* 10–13  *Post-transplant:* 13–26 | *Pre-transplant:* 13  *Post-transplant:* 15 | NR | | 13–16 | 14–16 |
| **Posterior wall thickness, mm**  **Range** | 15–17 | 10–24 | | 13–15 | 10–14 | | 11–15 | 9–11 | NR | | NR | | NR | |
| **LV diastolic diameter, mm**  **Range** | 44–54 | 31–45 | | 45–48 | 39–41 | | 42–54 | 47–54 | NR | | NR | | NR | |
| **LV mass index, g/m** | NR | | | NR | | | NR | | NR | | NR | | NR | |
| **Ejection fraction, %**  **Range** | NR | | | 25–50 | 40–60 | | 40–73 | 55–60 | NR | | NR | | NR | |
| **Atrial fibrillation, *n* (%)** | NR | | | NR | | | NR | | NR | | NR | | NR | |

**Table 5 A (*continued*)**

| **Characteristic** | **Russo 2012 [32]** | | **Satoskar 2011 [3]** | | **Tasaki 2013 [33]** | | **Vermeer 2017 [34]** | | **Yamamoto 2012 [7]** | | **Yazaki 2000 [35]** | |
| --- | --- | --- | --- | --- | --- | --- | --- | --- | --- | --- | --- | --- |
|  | **Men** | **Women** | **Men** | **Women** | **Men** | **Women** | **Men** | **Women** | **Men** | **Women** | **Men** | **Women** |
| **Sample size, *n* (%)** | 9 (90) | 1^b^ (10) | 1^b^ (50) | 1^b^ (50) | 9 (53) | 8 (47) | 3 (75) | 1^b^ (25) | 0 (0) | 2 (100) | 2 (33) | 4 (67) |
| **ATTR fibril composition, *n* (%)** | NR | | NR | | NR | | NR | | — | NR | NR | |
| **TTR genotype, *n* (%)**  **Val30Met**  **Val122Ile**  **Other** | 0 (0)  0 (0)  9 (100) | 0 (0)  0 (0)  1 (100) | 0 (0)  0 (0)  1 (100) | 0 (0)  0 (0)  1 (100) | 9 (100)  0 (0)  0 (0) | 8 (100)  0 (0)  0 (0) | 1 (33)  0 (0)  2 (67) | 0 (0)  1 (100)  0 (0) | — | NR | 2 (100)  0 (0)  0 (0) | 0 (0)  0 (0)  4 (100) |
| **Age, years**  **Range** | 71–83 | 85 | 77 | 66 | NR | | NR | | — | 56–74 | NR | |
| **Country** | Italy | | USA | | Japan | | the Netherlands | | — | Japan | Japan | |
| **Race/Ethnicity, *n* (%)**  **White**  **Black**  **Asian** | NR | | 1 (100)  0 (0)  0 (0) | 1 (100)  0 (0)  0 (0) | NR | 0 (0)  0 (0)  8 (100) | 3 (100)  0 (0)  0 (0) | 0 (0)  1 (100)  0 (0) | — | 0 (0)  0 (0)  2 (100) | 0 (0)  0 (0)  2 (33) | 0 (0)  0 (0)  4 (67) |
| **Carpal tunnel syndrome, *n* (%)** | 1 (11) | 1 (100) | NR | | NR | | 1 (33) | NR | — | NR | NR | |
| **NT-proBNP, pg/mL** | NR | | NR | | NR | | NR | | — | NR | NR | |
| **Aortic stenosis, *n* (%)** | NR | | NR | | NR | | NR | | — | NR | NR | |
| **Interventricular septal thickness, mm**  **Range** | NR | | NR | | NR | | NR | | — | 17–18 | NR | |
| **Posterior wall thickness, mm**  **Range** | NR | | NR | | NR | | 10–13 | 16 | — | 16–18 | NR | |
| **LV diastolic diameter, mm** | NR | | NR | | NR | | NR | | — | NR | NR | |
| **LV mass index, g/m** | NR | | NR | | NR | | NR | | — | NR | NR | |
| **Ejection fraction, %**  **Range** | NR | | NR | | NR | | NR | | — | 30–61 | NR | |
| **Atrial fibrillation, *n* (%)** | NR | | NR | | NR | | NR | | — | NR | NR | |

^a^Two studies are not included in this table as they did not report data for women in this category.

^b^Absolute values are reported in the rows below for the single individual in this subgroup.

*ATTR,* amyloid transthyretin; *ATTR-CM*, transthyretin amyloid cardiomyopathy*; LV*, left ventricular; *NR*, not reported; *NT-proBNP*, N-terminal pro-B-type natriuretic peptide; *TTR*, transthyretin

**B.** Cohort studies (*n* = 4)

| **Characteristic** | **Arvidsson 2015 [36]** | | **Lane 2019 [12]** | | **Rapezzi 2008 [37]** | | **Rapezzi 2009 [13]** | | |
| --- | --- | --- | --- | --- | --- | --- | --- | --- | --- |
|  | **Men** | **Women** | **Men** | **Women** | **Men** | **Women** | **Men** | | **Women** |
| **Sample size, *n* (%)** | 72 (67) | 35 (33) | 227 (70) | 96 (30) | 37 (71) | 15 (29) | 49 (80) | | 12 (20) |
| **ATTR fibril composition, *n* (%)** | Type A: 36 (50)  Type B: 36 (50) | Type A: 13 (37)  Type B: 22 (63) | NR | | NR | | NR | | |
| ***TTR* genotype, *n* (%)**  **Val30Met**  **Val122Ile**  **Other** | 72 (100)  0 (0)  0 (0) | 35 (100)  0 (0)  0 (0) | 0 (0)  146 (64)  81 (36) | 0 (0)  59 (61)  37 (39) | 12 (32)  0 (0)  25 (68) | 3 (20)  0 (0)  12 (80) | NR | | |
| **Age, years**  **Range**  **Mean (SD)** | Type A: 52–79 Type B: 31–76 | Type A: 56–86  Type B: 30–79 | NR | | NR | | 51.5 (12.3) | 75.5 (8.0) | |
| **Country** | Sweden | | UK | | Italy | | Italy | | |
| **Race/Ethnicity** | NR | | NR | | NR | | NR | | |
| **Carpal tunnel syndrome, *n* (%)** | NR | | NR | | NR | | NR | | |
| **NT-proBNP, pg/mL** | NR | | NR | | NR | | NR | | |
| **Aortic stenosis, *n* (%)** | NR | | NR | | NR | | NR | | |
| **Interventricular septal thickness, mm**  **Median (IQR)**  **Mean (SD)** | Type A: 18 (15–20) Type B: 12 (10–15) | Type A: 14* (12–16) Type B: 11 (10–13) | NR | | 15 (5) | 12* (3) | NR | | |
| **Posterior wall thickness, mm  Median (IQR)**  **Mean (SD)** | Type A: 12 (11–13) Type B: 10 (8–11)  — | Type A: 10* (9–10) Type B: 9 (8–10)  — | NR | | —  13 (4)^a^ | —  11 (3) | NR | | |
| **LV diastolic diameter, mm**  **Median (IQR)** | Type A: 49 (42–52) Type B: 48 (46–51) | Type A: 47 (39–50) Type B: 47 (44–50) | NR | | NR | | NR | | |
| **LV mass index, g/m**  **Mean (SD)** | NR | | NR | | 163 (66) | 125* (46) | NR | | |
| **Ejection fraction, %**  **Median (IQR)**  **Mean (SD)** | Type A: 64 (58–69) Type B: 63 (58–71)  — | Type A: 65 (58–74) Type B: 66 (57–71)  – | NR | | —  59 (10) | —  66* (12) | NR | | |
| **Atrial fibrillation, *n* (%)** | NR | | NR | | NR | | NR | | |

**P* < 0.05, for comparison of women vs. men.

*ATTR,* amyloid transthyretin; *ATTR-CM*, transthyretin amyloid cardiomyopathy*; IQR, interquartile range;* *LV*, left ventricular; *NR*, not reported; *NT-proBNP*, N-terminal pro-B-type natriuretic peptide; *SD*, standard deviation; *TTR*, transthyretin

**Table 6.** Demographic and clinical characteristics of patients with undefined ATTR-CM by patient sex in **(A)** case series and **(B)** cohort studies.

| **Characteristic** | 1. **Case series (*n* = 4^a^)** | | | | | | 1. **Cohort study (*n* = 1)** | |
| --- | --- | --- | --- | --- | --- | --- | --- | --- |
|  | **Galat 2016 [18]** | | **Jacobson 1997 [23]** | | **Wizenberg 1982 [38]** | | **Dungu 2014 [39]** | |
|  | **Men** | **Women** | **Men** | **Women** | **Men** | **Women** | **Men** | **Women** |
| **Sample size, *n* (%)** | 1^b^ (8) | 2 (67) | 15 (60) | 10 (40) | 6 (60) | 4 (40) | 45 (88) | 6 (12) |
| **Age, years**  **Range** | NR | | 60–97 | 64–85 | 59–85 | 67–84 | NR | |
| **Country** | France | | USA | | USA | | UK | |
| **Race/Ethnicity, %**  **White**  **Black**  **Asian** | NR | | 0 (0)  15 (100)  0 (0) | 0 (0)  10 (100)  0 (0) | NR | | NR | |
| **Carpal tunnel syndrome, *n* (%)** | 0 (0) | 0 (0) | NR | | NR | | NR | |
| **NT-proBNP, pg/mL**  **Range** | 2425 | 1932–2799 | NR | | NR | | NR | |
| **Aortic stenosis, *n* (%)** | 1 (100) | 1 (50) | NR | | NR | | NR | |
| **Interventricular septal thickness, mm**  **Range**  **Mean (SD)** | 22  — | 13–14  — | NR | | —  16 (2) | —  17 (2) | NR | |
| **Posterior wall thickness, mm**  **Mean (SD)** | NR | | NR | | 16 (2) | 17 (2) | NR | |
| **LV diastolic diameter, mm**  **Mean (SD)** | NR | | NR | | 54 (9) | 46 (10) | NR | |
| **LV mass index, g/m** | NR | | NR | | NR | | NR | |
| **Ejection fraction, %**  **Range**  **Mean (SD)** | 55  —  — | 56–69  — | NR | | NR | | —  55 (12) | —  62 (14) |
| **Atrial fibrillation, *n* (%)** | 0 (0) | 1 (50) | 1 (7) | 1 (10) | NR | | NR | |

^a^One study is not included in this table as it did not report data for women in this category.

^b^Absolute values are reported in the rows below for the single individual in this subgroup.

*ATTR-CM*, transthyretin amyloid cardiomyopathy*;* *LV*, left ventricular; *NR*, not reported; *NT-proBNP*, N-terminal pro-B-type natriuretic peptide; *SD*, standard deviation

**References**

1. Dorbala S, Ando Y, Bokhari S, Dispenzieri A, Falk RH, Ferrari VA, Fontana M, Gheysens O, Gillmore JD, Glaudemans A, Hanna MA, Hazenberg BPC, Kristen AV, Kwong RY, Maurer MS, Merlini G, Miller EJ, Moon JC, Murthy VL, Quarta CC, Rapezzi C, Ruberg FL, Shah SJ, Slart R, Verberne HJ, Bourque JM (2019) ASNC/AHA/ASE/EANM/HFSA/ISA/SCMR/SNMMI Expert consensus recommendations for multimodality imaging in cardiac amyloidosis: part 2 of 2-diagnostic criteria and appropriate utilization. J Card Fail 25(11):854–865. doi:10.1016/j.cardfail.2019.08.002

2. Helder MRK, Schaff HV, Nishimura RA, Gersh BJ, Dearani JA, Ommen SR, Mereuta OM, Theis JD, Dogan A, Edwards WD (2014) Impact of incidental amyloidosis on the prognosis of patients with hypertrophic cardiomyopathy undergoing septal myectomy for left ventricular outflow tract obstruction. Am J Cardiol 114(9):1396–1399

3. Satoskar AA, Efebera Y, Hasan A, Brodsky S, Nadasdy G, Dogan A, Nadasdy T (2011) Strong transthyretin immunostaining: potential pitfall in cardiac amyloid typing. Am J Surg Pathol 35(11):1685–1690. doi:10.1097/PAS.0b013e3182263d74

4. Sharma PP, Payvar S, Litovsky SH (2008) Histomorphometric analysis of intramyocardial vessels in primary and senile amyloidosis: epicardium versus endocardium. Cardiovasc Pathol 17(2):65–71

5. Treibel TA, Fontana M, Gilbertson JA, Castelletti S, White SK, Scully PR, Roberts N, Hutt DF, Rowczenio DM, Whelan CJ, Ashworth MA, Gillmore JD, Hawkins PN, Moon JC (2016) Occult transthyretin cardiac amyloid in severe calcific aortic stenosis. Circ Cardiovasc Imaging 9(8):e005066

6. Xu B, Godoy Rivas C, Rodriguez ER, Tan C, Gillinov AM, Harb S, Jellis C, Griffin B (2019) Unrecognized cardiac amyloidosis at the time of mitral valve surgery: incidence and outcomes. Cardiology 142(4):253–258. doi:10.1159/000499933

7. Yamamoto Y, Onoguchi M, Haramoto M, Kodani N, Komatsu A, Kitagaki H, Tanabe K (2012) Novel method for quantitative evaluation of cardiac amyloidosis using ^201^Tl-Cl and ^99m^Tc-PYP SPECT. Ann Nucl Med 26(8):634–643

8. Zegri-Reiriz I, de Haro-Del Moral FJ, Dominguez F, Salas C, de la Cuadra P, Plaza A, Krsnik I, Gonzalez-Lopez E, Garcia-Pavia P (2019) Prevalence of cardiac amyloidosis in patients with carpal tunnel syndrome. J Cardiovasc Transl Res 12(6):507–513. doi:10.1007/s12265-019-09895-0

9. aus dem Siepen F, Bauer R, Voss A, Hein S, Aurich M, Riffel J, Mereles D, Rocken C, Buss SJ, Katus HA, Kristen AV (2018) Predictors of survival stratification in patients with wild-type cardiac amyloidosis. Clin Res Cardiol 107(2):158–169

10. Gonzalez-Lopez E, Gagliardi C, Dominguez F, Quarta CC, De Haro-Del Moral FJ, Milandri A, Salas C, Cinelli M, Cobo-Marcos M, Lorenzini M, Lara-Pezzi E, Foffi S, Alonso-Pulpon L, Rapezzi C, Garcia-Pavia P (2017) Clinical characteristics of wild-type transthyretin cardiac amyloidosis: disproving myths. Eur Heart J 38(24):1895–1904

11. Kim D, Lee GY, Choi JO, Kim K, Kim SJ, Jeon ES (2019) Associations of electrocardiographic parameters with left ventricular longitudinal strain and prognosis in cardiac light chain amyloidosis. Sci Rep 9(1):7746

12. Lane T, Fontana M, Martinez-Naharro A, Quarta CC, Whelan CJ, Petrie A, Rowczenio DM, Gilbertson JA, Hutt DF, Rezk T, Strehina SG, Caringal-Galima J, Manwani R, Sharpley FA, Wechalekar AD, Lachmann HJ, Mahmood S, Sachchithanantham S, Drage EPS, Jenner HD, McDonald R, Bertolli O, Calleja A, Hawkins PN, Gillmore JD (2019) Natural history, quality of life, and outcome in cardiac transthyretin amyloidosis. Circulation 140(1):16–26

13. Rapezzi C, Merlini G, Quarta CC, Riva L, Longhi S, Leone O, Salvi F, Ciliberti P, Pastorelli F, Biagini E, Coccolo F, Cooke RM, Bacchi-Reggiani L, Sangiorgi D, Ferlini A, Cavo M, Zamagni E, Fonte ML, Palladini G, Salinaro F, Musca F, Obici L, Branzi A, Perlini S (2009) Systemic cardiac amyloidoses: disease profiles and clinical courses of the 3 main types. Circulation 120(13):1203–1212

14. Abulizi M, Cottereau AS, Guellich A, Vandeventer S, Galat A, Van Der Gucht A, Planté-Bordeneuve V, Dubois-Rande JL, Bodez D, Rosso J, Damy T, Itti E (2018) Early-phase myocardial uptake intensity of ^99m^Tc-HMDP vs ^99m^Tc-DPD in patients with hereditary transthyretin-related cardiac amyloidosis. J Nucl Cardiol 25(1):217–222

15. Choi K, Seok JM, Kim BJ, Choi YC, Shin HY, Sunwoo IN, Kim DS, Sung JJ, Lee GY, Jeon ES, Kim NH, Min JH, Oh J (2018) Characteristics of South Korean patients with hereditary transthyretin amyloidosis. J Clin Neurol (Korea) 14(4):537–541

16. Damy T, Costes B, Hagege AA, Donal E, Eicher JC, Slama M, Guellich A, Rappeneau S, Gueffet JP, Logeart D, Planté-Bordeneuve V, Bouvaist H, Huttin O, Mulak G, Dubois-Rand JL, Goossens M, Canoui-Poitrine F, Buxbaum JN (2016) Prevalence and clinical phenotype of hereditary transthyretin amyloid cardiomyopathy in patients with increased left ventricular wall thickness. Eur Heart J 37(23):1826–1834

17. Di Bella G, Minutoli F, Mazzeo A, Vita G, Oreto G, Carerj S, Anfuso C, Russo M, Gaeta M (2010) MRI of cardiac involvement in transthyretin familial amyloid polyneuropathy. AJR Am J Roentgenol 195(6):W394–399. doi:10.2214/AJR.09.3721

18. Galat A, Guellich A, Bodez D, Slama M, Dijos M, Zeitoun DM, Milleron O, Attias D, Dubois-Rande JL, Mohty D, Audureau E, Teiger E, Rosso J, Monin JL, Damy T (2016) Aortic stenosis and transthyretin cardiac amyloidosis: the chicken or the egg? Eur Heart J 37(47):3525–3531

19. Gustafsson S, Ihse E, Henein MY, Westermark P, Lindqvist P, Suhr OB (2012) Amyloid fibril composition as a predictor of development of cardiomyopathy after liver transplantation for hereditary transthyretin amyloidosis. Transplantation 93(10):1017–1023

20. Haagsma EB, Van Gameren II, Bijzet J, Posthumus MD, Hazenberg BPC (2007) Familial amyloidotic polyneuropathy: long-term follow-up of abdominal fat tissue aspirate in patients with and without liver transplantation. Amyloid 14(3):221–226

21. Hattori T, Takei Y, Koyama J, Nakazato M, Ikeda S (2003) Clinical and pathological studies of cardiac amyloidosis in transthyretin type familial amyloid polyneuropathy. Amyloid 10(4):229–239

22. Ihse E, Rapezzi C, Merlini G, Benson MD, Ando Y, Suhr OB, Ikeda SI, Lavatelli F, Obici L, Quarta CC, Leone O, Jono H, Ueda M, Lorenzini M, Liepnieks J, Ohshima T, Tasaki M, Yamashita T, Westermark P (2013) Amyloid fibrils containing fragmented ATTR may be the standard fibril composition in ATTR amyloidosis. Amyloid 20(3):142–150

23. Jacobson DR, Pastore RD, Yaghoubian R, Kane I, Gallo G, Buck FS, Buxbaum JN (1997) Variant-sequence transthyretin (isoleucine 122) in late-onset cardiac amyloidosis in black Americans. N Engl J Med 336(7):466–473

24. Kero T, Sorensen J, Antoni G, Wilking H, Carlson K, Vedin O, Rosengren S, Wikstrom G, Lubberink M (2020) Quantification of (11)C-PIB kinetics in cardiac amyloidosis. J Nucl Cardiol 27(3):774-784. doi:10.1007/s12350-018-1349-x

25. Koike H, Hashimoto R, Tomita M, Kawagashira Y, Iijima M, Tanaka F, Sobue G (2011) Diagnosis of sporadic transthyretin Val30Met familial amyloid polyneuropathy: a practical analysis. Amyloid 18(2):53–62

26. Morner S, Hellman U, Suhr OB, Kazzam E, Waldenstrom A (2005) Amyloid heart disease mimicking hypertrophic cardiomyopathy. J Intern Med 258(3):225–230

27. Nelson WW, Choi JC, Vanderpoel J, Damaraju CV, Wildgoose P, Fields LE, Schein JR (2013) Impact of co-morbidities and patient characteristics on international normalized ratio control over time in patients with nonvalvular atrial fibrillation. Am J Cardiol 112(4):509–512

28. Nelson LM, Gustafsson F, Gimsing P (2015) Characteristics and long-term outcome of patients with systemic immunoglobulin light-chain amyloidosis. Acta Haematol 133(4):336–346

29. Okamoto S, Yamashita T, Ando Y, Ueda M, Misumi Y, Obayashi K, Horibata Y, Uchino M (2008) Evaluation of myocardial changes in familial amyloid polyneuropathy after liver transplantation. Intern Med 47(24):2133–2137. doi:10.2169/internalmedicine.47.1399

30. Oshima T, Kawahara S, Ueda M, Kawakami Y, Tanaka R, Okazaki T, Misumi Y, Obayashi K, Yamashita T, Ohya Y, Ihse E, Shinriki S, Tasaki M, Jono H, Asonuma K, Inomata Y, Westermark P, Ando Y (2014) Changes in pathological and biochemical findings of systemic tissue sites in familial amyloid polyneuropathy more than 10 years after liver transplantation. J Neurol Neurosurg Psychiatry 85(7):740–746

31. Pilebro B, Arvidsson S, Lindqvist P, Sundstrom T, Westermark P, Antoni G, Suhr O, Sorensen J (2018) Positron emission tomography (PET) utilizing Pittsburgh compound B (PIB) for detection of amyloid heart deposits in hereditary transthyretin amyloidosis (ATTR). J Nucl Cardiol 25(1):240–248

32. Russo M, Mazzeo A, Stancanelli C, Di Leo R, Gentile L, Di Bella G, Minutoli F, Baldari S, Vita G (2012) Transthyretin-related familial amyloidotic polyneuropathy: description of a cohort of patients with Leu64 mutation and late onset. J Peripher Nerv Syst 17(4):385–390

33. Tasaki M, Ueda M, Obayashi K, Koike H, Kitagawa K, Ogi Y, Jono H, Su Y, Suenaga G, Oshima T, Misumi Y, Yoshida M, Yamashita T, Sobue G, Ando Y (2013) Effect of age and sex differences on wild-type transthyretin amyloid formation in familial amyloidotic polyneuropathy: a proteomic approach. Int J Cardiol 170(1):69–74

34. Vermeer AMC, Janssen A, Boorsma PC, Mannens M, Wilde AAM, Christiaans I (2017) Transthyretin amyloidosis: a phenocopy of hypertrophic cardiomyopathy. Amyloid 24(2):87–91. doi:10.1080/13506129.2017.1322573

35. Yazaki M, Tokuda T, Nakamura A, Higashikata T, Koyama J, Higuchi K, Harihara Y, Baba S, Kametani F, Ikeda S (2000) Cardiac amyloid in patients with familial amyloid polyneuropathy consists of abundant wild-type transthyretin. Biochem Biophys Res Commun 274(3):702–706. doi:10.1006/bbrc.2000.3203

36. Arvidsson S, Pilebro B, Westermark P, Lindqvist P, Suhr OB (2015) Amyloid cardiomyopathy in hereditary transthyretin V30M amyloidosis - impact of sex and amyloid fibril composition. PLoS One 10(11):e0143456. doi:10.1371/journal.pone.0143456

37. Rapezzi C, Riva L, Quarta CC, Perugini E, Salvi F, Longhi S, Ciliberti P, Pastorelli F, Biagini E, Leone O, Cooke RM, Bacchi-Reggiani L, Ferlini A, Cavo M, Merlini G, Perlini S, Pasquali S, Branzi A (2008) Gender-related risk of myocardial involvement in systemic amyloidosis. Amyloid 15(1):40–48. doi:10.1080/13506120701815373

38. Wizenberg TA, Muz J, Sohn YH, Samlowski W, Weissler AM (1982) Value of positive myocardial technetium-99m-pyrophosphate scintigraphy in the noninvasive diagnosis of cardiac amyloidosis. Am Heart J 103(4 Pt 1):468–473. doi:10.1016/0002-8703(82)90331-3

39. Dungu JN, Valencia O, Pinney JH, Gibbs SD, Rowczenio D, Gilbertson JA, Lachmann HJ, Wechalekar A, Gillmore JD, Whelan CJ, Hawkins PN, Anderson LJ (2014) CMR-based differentiation of AL and ATTR cardiac amyloidosis. JACC Cardiovasc Imaging 7(2):133–142. doi:10.1016/j.jcmg.2013.08.015
